# MicroRNA 211-5p inhibits cancer cell proliferation and migration in pancreatic cancer by targeting BMP2

**DOI:** 10.18632/aging.205320

**Published:** 2023-12-06

**Authors:** Dan Li, Chen Luo, Jianyong Deng, Yongkang Xu, Shumin Fu, Kan Liu, Jianbing Wu

**Affiliations:** 1 Department of Gastroenterology, The Second Affiliated Hospital of Nanchang University, Nanchang, Jiangxi Province, China; 2 Department of Oncology, The Second Affiliated Hospital of Nanchang University, Nanchang, Jiangxi Province, China; 3 Department of General Surgery, The First Affiliated Hospital of Nanchang University, Nanchang, Jiangxi Province, China; 4 Jiangxi Province Medical College of Nanchang University, Nanchang, Jiangxi Province, China; 5 Department of General Surgery, Third Affiliated Hospital of Nanchang University, Nanchang, Jiangxi Province, China

**Keywords:** miR-211-5p, pancreatic cancer, BMP2, proliferation, metastasis

## Abstract

MicroRNAs (miRNAs) are essential to the tumour growth and metastasis of several cancers. However, the implied functions of miR-211-5p in pancreatic cancer (PC) remains poorly known. In the present study, we discovered that miR-211-5p was a significantly downregulated miRNA in PC tissues compared to adjacent non-tumour tissues. Moreover, we revealed that miR-211-5p overexpression suppressed the proliferation and metastasis of PC cells. Mechanistically, miR-211-5p directly bond to 3'UTR of bone morphogenetic protein-2 (BMP2) and negatively regulated its expression. Rescue experiments showed that the biological function of miR-211-5p was reversed by BMP-2 overexpression in PC cells. Clinical data indicated that BMP2 expression was negatively correlated with miR-211-5p levels in PC patients. Our study provided evidence that miR-211-5p served as a significant suppressor in PC, provided potential targets for prognosis and treatment of patients with PC.

## INTRODUCTION

Pancreatic cancer (PC) is the worst prognosis malignancy within the category of digestive system tumors, known for its aggressive nature and significant mortality rate [1, 2]. While significant advancements have been made in the management of pancreatic cancer, a majority of patients experience postoperative recurrence due to local infiltration or extensive metastasis [3, 4]. Due to the advanced stage of PC in the majority of diagnosed patients, it is not feasible to perform a comprehensive radical operation for PC patients. Hence, there exists a significant requirement to discover efficacious treatment options and preventive strategies against metastasis and recurrence, aiming at enhancing the overall management of PC.

MicroRNAs (miRNAs), the regulators mentioned are highly regarded as crucial factors in tumor development and possess significant biomarker and diagnostic potential [5–7]. Recently, a growing body of research has revealed that the role of miRNAs as tumor suppressor genes is evident in various types of tumors. The malignant progression of PC cells is regulated by miR-939-5p through targeting ARHGAP4 in PC [8]. MiR-211 has been confirmed to be associated with pancreatic cancer [9]. MiRNAs play a pivotal role in orchestrating cancer development by modulating key cellular processes, including apoptosis, cell proliferation, migration, and invasion [10–12]. MiR-211-5p is a highly conserved microRNA, exhibits remarkable conservation across species, which is involved in the process of tumorigenesis and development in different cancers [13–15]. MiR-211-5p was recently characterized as a tumor suppressor in prostate cancer [16]. Nevertheless, the mechanisms underlying miR-211-5p in PC remains to be investigated.

Bone morphogenetic protein 2 (BMP2), belongs to the transforming growth factors-β (TGF-β) subfamily, was initially discovered for its ability to stimulate osteogenesis and facilitate the growth of bone tissue [17, 18]. The aberrant expression of BMP2 has been demonstrated to participate in the biological process of outside the skeletal system, including regulating the proliferation, differentiation, processes of cancer cells [19]. The most recent research results have revealed that the promotion of epithelial mesenchymal transition (EMT) and enhancement of tumor cells’ metastatic capability are facilitated by BMP2 [20]. Moreover, evidence has demonstrated that BMP2 is upregulated in many cancers, and is related to the progression and growth of malignant tumors including non-small-cell lung cancer [21], osteosarcoma [22], gastric cancer [23], and nasopharyngeal carcinoma [24]. The complete elucidation of the regulatory mechanism of BMP2 in PC necessitates further investigation.

The preliminary results of this investigation revealed a significant reduction in the levels of miR-211-5p observed in both PC specimens and cells. Furthermore, when the levels of miR-211-5p was increased, it inhibited the proliferative ability of PC, as evidenced through the CCK-8 assay. The transwell assay revealed a notable decrease in the migratory capacity of PC cells upon upregulation of miR-211-5p expression. Additionally, according to the luciferase reporter assay, it was observed that BMP2 expression is directly suppressed by miR-211-5p specific binding to the 3’-UTR region of BMP2. Further rescue experiments demonstrate that the proliferation and migration of PC are suppressed by miR-211-5p through its targeting of BMP2. The function of the MiR-211-5p/BMP2 axis in PC development highlights its promising target for therapeutic interventions of PC.

## MATERIALS AND METHODS

### Patient samples

All PC samples and their corresponding adjacent samples were obtained from patients who underwent surgeries at the Second Affiliated Hospital of Nanchang University between January 2015 and January 2017. All samples were stored at a temperature of -80° C after removal. The Ethics Committee of the second affiliated Hospital of Nanchang University granted approval for our research.

### Cell culture

The human normal pancreatic cell line (HPDE6-C7) and PC cell lines (PANC-1, AsPC-1, BxPC-3, CFPAC-3, SW1990) were purchased from the esteemed Shanghai Institute of Cells under the auspices of the Chinese Academy of Sciences. All cells were cultured in DMEM (Gibco; Thermo Fisher Scientific, USA) with 10% FBS (Gibco; Thermo Fisher Scientific, USA) in a 37° C with 5% CO2. MiR-NC mimics and miR-211-5p mimics were synthesized by Ribo Bio (Guangzhou, China). Small interfering RNAs (siRNAs) specific for BMP2 (siBMP2) and pcDNA 3.1(+)-BMP2 were used as described previously [18].

### The prediction of miRNA targets

The potential gene targeted by miR-211-5p was predicted using TargetScan (https://www.Targetscan.org/). Additionally, it was employed to explore the alleged interaction site between them.

### Cell counting kit-8 (CCK-8) assay

PC cells were harvested 48 hours post-transfection and evenly distributed at a seeding density of 2000 cells per well. Following that, PC cells were different time intervals: 24h, 48h, 72h, and 96h. Subsequently, each well was supplemented with a volume of CCK-8 solution (Dojindo Molecular Technologies, Kumamoto, Japan) measuring only 10 μL and incubated for a duration of 2 hours. The optical density (OD) value of each hole was measured by a microplate reader set to an absorption wavelength of 490 nm.

### Transwell assay

The transwell chamber was seeded with PC cells at a density of 1x10^4^ cells per group. Following 24 hours, subependymal cells were fixed using formaldehyde, and a staining solution consisting of 0.2% crystal violet was applied.

### Western blotting

As mentioned earlier, western blotting analysis used as previously described [19]. The antibodies examined in this investigation encompass the BMP2 rabbit antibody (ab53146, 1:1000, Abcam, USA), mouse antibody GAPDH (ab8245, 1:3000, Abcam, USA).

### Quantitative real-time PCR (qRT-PCR)

The RNA extraction process involved the utilization of the RNAiso Plus reagent (Takara, Japan) on either tissue samples or cultured cells. Subsequently, reverse transcription was performed on the extracted RNA to generate complementary DNA (cDNA), which was subsequently employed for polymerase chain reaction (PCR) amplification. The primers were as follows: miR-211-5p (F): 5’-ACACTCCAGCTGGGCAAGTAGCATCAACTA-3’, miR-211-5p (R): 5’-TGGTGTCGTGGAGTCG-3’, U6 (F): 5’-CTCGCTTCGGCAGCACA-3’ and U6 (R): 5’-AACGCTTCACGAATTTGCGT-3’, BMP2 (F): 5’-GGTATGCTCCTGCCGCCTGCA-3’ and BMP2 (R): 5’-ATCAGCATAGGCCGGTGCAA-3’, GAPDH (F): 5’-CAAGGTCATCCATGACAACTTTG-3’, and GAPDH (R): 5’-GTCCACCACCCTGTTGCTGTAG-3’. The ABI Step One software (Applied Biosystems, Foster City, CA, USA) was for data analysis, while the 2^-^*^ΔΔCt^* method was employed to analyze the relative expression levels of mRNA.

### Dual-luciferase reporter system

The pmirGLO vector 3’-UTR was utilized to construct the wild-type (Wt) and mutant (Mut) BMP2, which were synthesized by Shanghai GenePharma, China. The cells were transfected with miR-NC mimics or miR-211-5p-mimics at a concentration of 20 nM. Following a 48-hour incubation period, the cell luciferase activity was assessed using the dual-luciferase reporting system from Promega (Madison, WI, USA). The experimental procedure followed the previously described methodology [25].

### Xenograft experiments

The *in vivo* xenograft model was established using PC cells that were transfected with either miR-NC mimic or miR-211-5p mimic. These cells were then randomly divided into two groups. Depending on the grouping, a dose of 5×10^6^ miR-NC mimics or miR-211-5p mimics suspended in a mixture of 0.1 mL PBS and 0.1 mL Matrigel (BD Biosciences, San Jose, CA, USA) was subcutaneously injected into the right axilla of each mouse.

### Statistical analysis

The analysis of the data was conducted using SPSS 22.0 software (Chicago, IL, USA), and all conclusions were based on this examination. GraphPad Prism 8.0. The significance of differences between two and multiple groups was assessed through One-way ANOVA and Student’s t-test. *p <* 0.05 was considered statistically significant.

### Consent for publication

All authors agree for publication.

## RESULTS

### The expression levels of miR-211-5p exhibited a significant reduction in both pancreatic cancer (PC) tissues and cells

**Figure 1:**
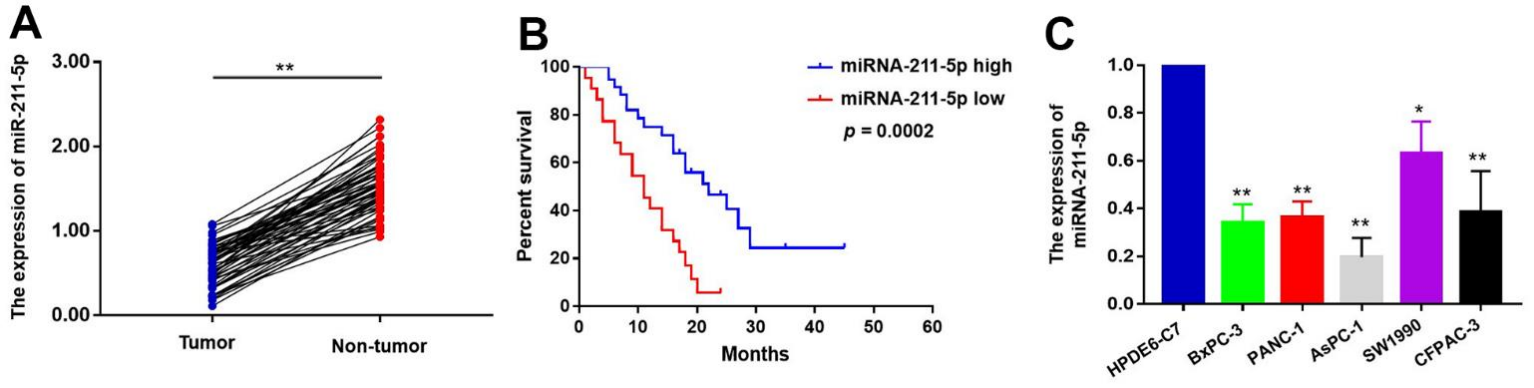
**The expression levels of miR-211-5p in PC tissues and cells.** (**A**) The expression of miR-211-5p was detected by qRT-PCR between PC tissues and corresponding adjacent tissues (n=58, *p* < 0.001, Wilcoxon signed-rank test). GAPDH was used as a loading control. Tumor represented PC tissues and non-tumor represented corresponding adjacent tissues. (**B**) Kaplan–Meier survival curve of the relationship between miR-211-5p high and low expression groups and the prognosis of PC patients (n=58, *p*=0.0002, Log-rank test). (**C**) The expression of miR-211-5p in HPDEC, PANC-1, AsPC-1, SW1990, BxPC-3 and CFPAC-1 cells analyzed by qRT-PCR. Data represent the mean ± SD of triplicate experiments and were statistically analyzed with Student’s t-test, *p < 0.05, ** *p* < 0.01.

The suppressive impact of miR-211-5p on neoplastic cell proliferation has been extensively documented across various human malignancies. To investigate the function of miR-211-5p in pancreatic cancer (PC), we first examined the levels of miR-211-5p between PC tissues and their adjacent tissues. qRT-PCR assay indicated that miR-211-5p was highly expressed in corresponding adjacent tissues compared to PC tissues (Figure 1A). The clinicopathological correlation analysis revealed a significant association between the levels of miR-211-5p and TNM stage (Table 1, *p* = 0.023). In addition, a significant correlation was observed between the survival of patients with pancreatic cancer and the levels of miR-211-5p, as determined by Kaplan-Meier survival analysis. (Figure 1B, *p* = 0.0002). Subsequently, the analysis was conducted on the levels of miR-211-5p in PC cells. Our findings indicated a decrease in the levels of miR-211-5p in ASPC-1, BXPC-3, and PANC-1 cells when compared to their normal cell (Figure 1C). Our findings showed that miR-211-5p served as a promising prognostic biomarker for individuals diagnosed with PC.

**Table 1:** The relationship between miR-211-5p expression and clinicopathological characteristics in 58 PC patients.

**Characteristics**	**No. of patients**	**miR-211-5p expression**		***p*-value**
		**High n=37**	**Low n=21**	
Age (years)				0.601
<60	25	15	10	
≥60	33	22	11	
Gender				0.702
Male	24	16	8	
Female	34	21	13	
Lymph node metastasis				0.732
Negative	33	23	14	
Positive	25	14	7	
TNM stage				**0.023**
I and II	36	27	9	
III and IV	22	10	12	

Bold values are statistically significant, *p* < 0.05.

### The *in vitro* experiments demonstrated that the upregulation of miR-211-5p resulted in a decrease in the proliferation and metastatic potential of PC cells

**Figure 2:**
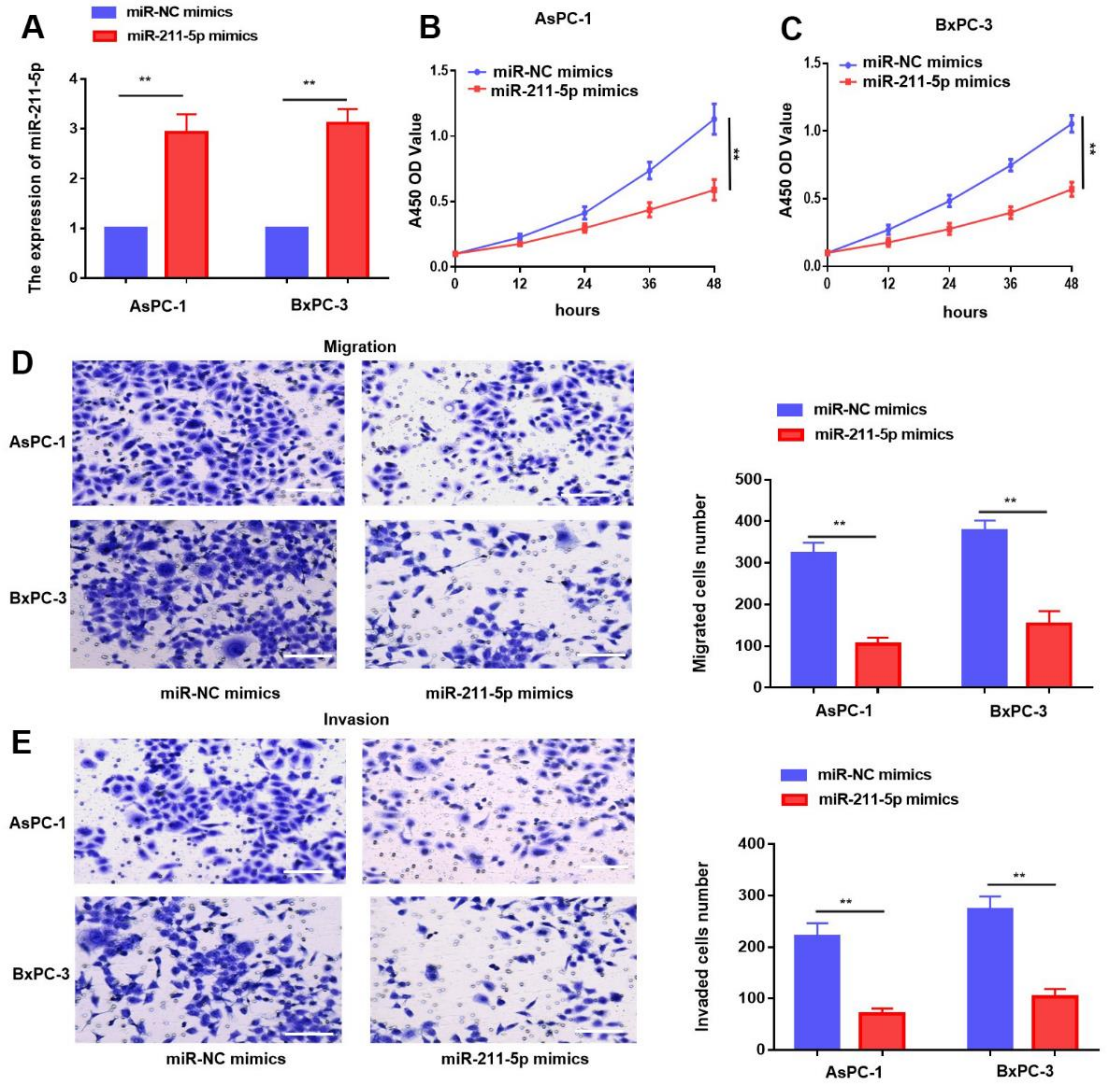
**Overexpression of miR-211-5p inhibited cell proliferation and metastasis in PC *in vitro*.** (**A**) miR-211-5p expression was analyzed in AsPC-1 and BxPC-3 cells transient transfected with miR-NC mimics, and miR-211-5p mimics by qRT-PCR. (**B**, **C**) CCK-8 analysis with miR-211-5p mimics or miR-NC mimics in AsPC-1 and BxPC-3. (**D**, **E**) Transwell assay for cell migration with miR-211-5p mimics or miR-NC mimics in AsPC-1 and BxPC-3. **p < 0.01, scale bar, 100 μm.

The observed expression pattern and potential clinical significance of miR-211-5p indicate its possible inhibitory role in PC. To explore the impact of miR-211-5p on PC cells, we introduced miR-NC mimics and miR-211-5p mimics into PC cells through transfection. The qRT-PCR assay demonstrated a notable increase in the levels of miR-211-5p in both AsPC-1 and BxPC-3 cells (Figure 2A). Moreover, the proliferation ability of PC cells was assessed using the CCK-8 assay. Our findings indicated that the enhanced the levels of miR-211-5p exerted a substantial suppressive effect on cellular proliferation in both AsPC-1 and BxPC-3 cell lines (Figure 2B, 2C). Similarly, transwell assay demonstrated that cell metastasis was inhibited by the upregulation of miR-211-5p in AsPC-1 and BxPC-3 cells (Figure 2D, 2E). Our findings suggested that miR-211-5p exerted a suppressive effect on the cellular proliferation and metastasis of PC.

### *In vivo*, the upregulation of miR-211-5p resulted in the suppression of tumor development

**Figure 3:**
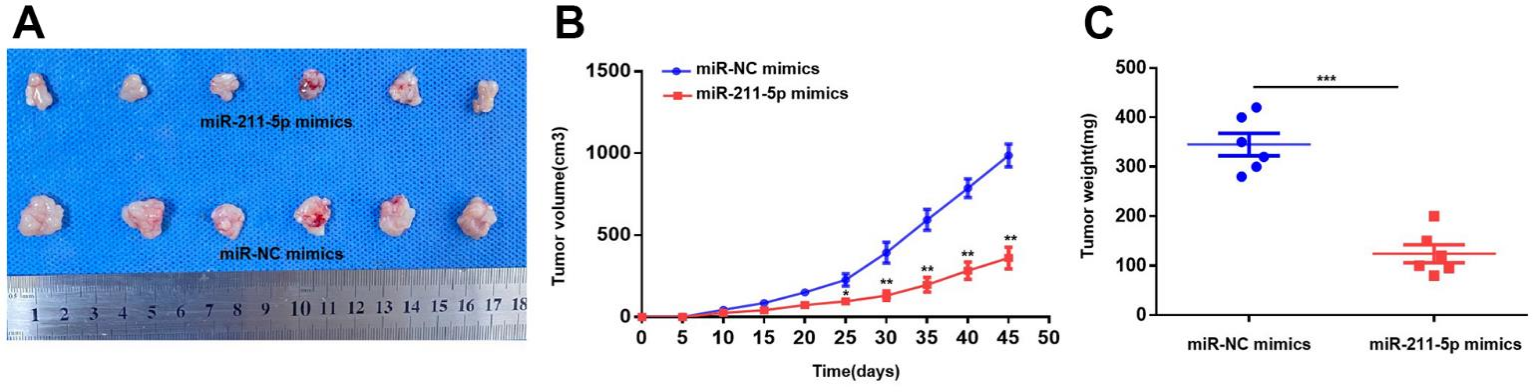
**Overexpression of miR-211-5p inhibits tumor proliferation *in vivo*.** (**A**) The images of tumors the miR-NC mimics and miR-211-5p mimics groups. (**B**) Comparison of tumor volume of the two groups every 5 days. (**C**) Comparison of tumor weight of the two groups 45 days after the subcutaneous injection. *p < 0.05, **p < 0.01.

To evaluate the function of miR-211-5p on PC *in vivo*, we injected intravenous injections of PC cells that were transfected with miR-NC mimics and miR-211-5p mimics to BALB/c nude mice (male, 5 weeks). The size of the tumors injected with cells transfected with miR-211-5p mimics was found to be significantly smaller compared to those in the group of mice treated with miR-NC mimics, as indicated by the study (Figure 3A). Moreover, the miR-211-5p mimics group exhibited significantly reduced tumor weight and volume compared to the miR-NC mimics group (Figure 3B, 3C). Altogether, the significant inhibition of *in vivo* PC growth was observed upon the upregulation of miR-211-5p.

### BMP2 was identified as a direct target of miR-211-5p

**Figure 4:**
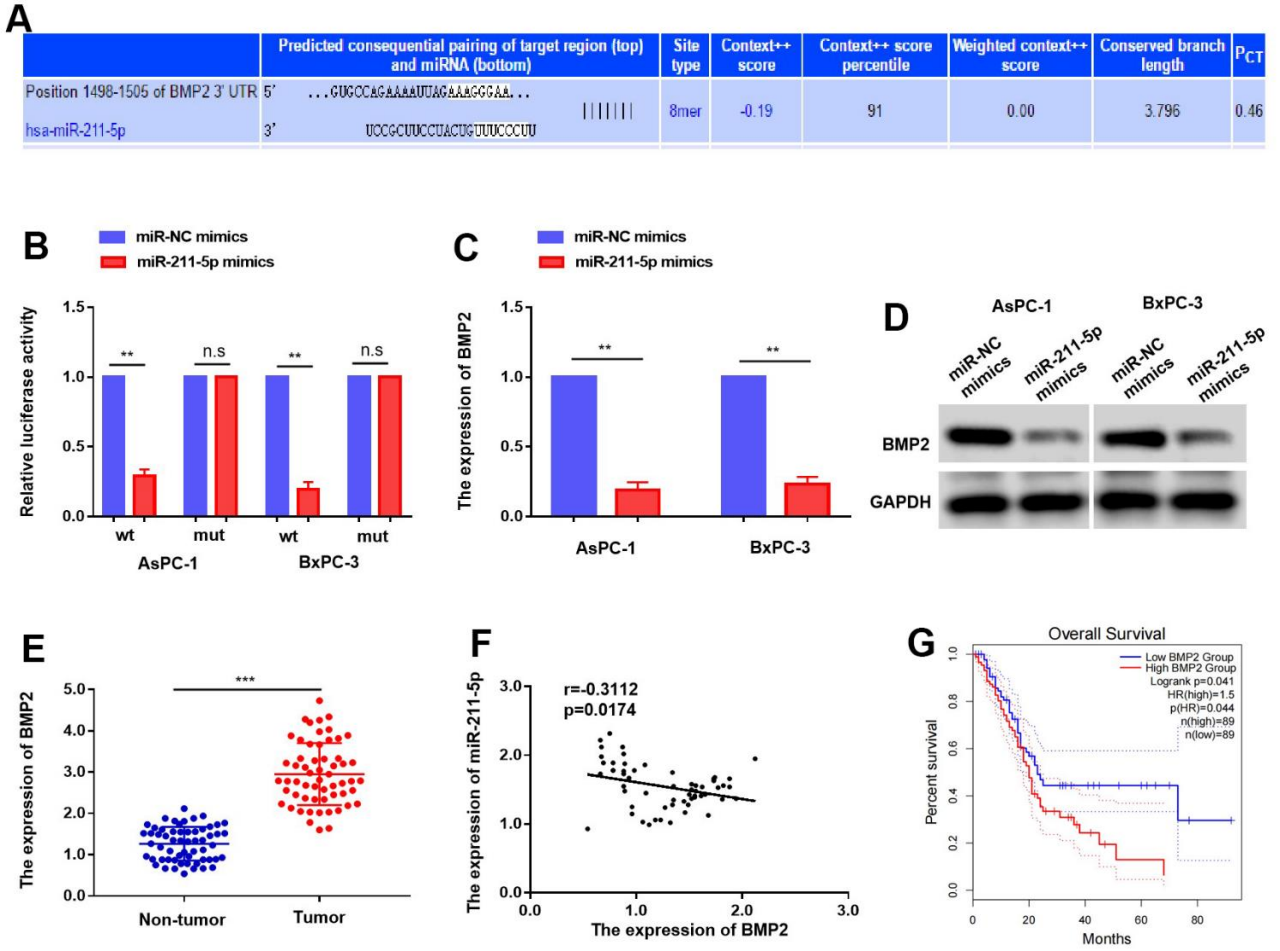
**Targeting of BMP2 by miR-211-5p in PC.** (**A**) BMP2 was predicted as a potential targets of miR-211-5p. (**B**) The luciferase activity of BMP2 WT or MUT’3 level of BMP2 affected with miR-211-5p mimics in AsPC-1 and BxPC-3. (**C**, **D**) the expression mRNA and protein of BMP2 with miR-211-5p mimics in AsPC-1 and BxPC-3 cells tested by qRT-PCR and western blot. (**E**) The expression of BMP2 mRNA is upregulated in PC tissues. (**F**) The relationship of BMP2 and miR-211-5p in PC. (r = −0.3112, *p* = 0.0174). (**G**) The overall survival time of PC patients with different expression of BMP2.

To explore the mechanism responsible for the inhibitory function of miR-211p, we employed the online platform TargetScan to predict BMP2 as a potential target of miR-211-5p (Figure 4A). Notably, the experiment involving the dual luciferase reporter assay was performed, and as expected, the miR-211-5p-mimics demonstrated inhibition of relative luciferase activity in AsPC-1 and BxPC-3 cells by targeting the BMP2 3’ UTR-WT (Figure 4B). Furthermore, our findings revealed that the upregulation of miR-211-5p decreased the levels of BMP2 mRNA and protein in AsPC-1 and BxPC-3 cells (Figure 4C, 4D). To further explore their correlation in clinical specimens, we evaluated the expression levels of BMP2 in 58 sets of tumor and non-tumor pancreatic cancer tissues. In line with prior discoveries, the expression of BMP2 in PC samples was found to be significantly elevated compared to non-tumor PC tissues (Figure 4E). Moreover, there was a negative correlation observed between the levels of BMP2 and miR-211-5p in PC samples (Figure 4F, r=-0.3112, *p* = 0.0174). Furthermore, the GEPIA (Gene Expression Profiling Interactive Analysis) findings revealed a significant association between increased BMP2 levels and decreased survival rates among PC patients, as indicated by the Kaplan-Meier survival analysis (Figure 4F, *p* = 0.041). This evidence verified that BMP2 was a direct target of miR-211-5p in PC.

### Upregulation of BMP2 enhances the proliferative and metastatic potential of PC cells

**Figure 5:**
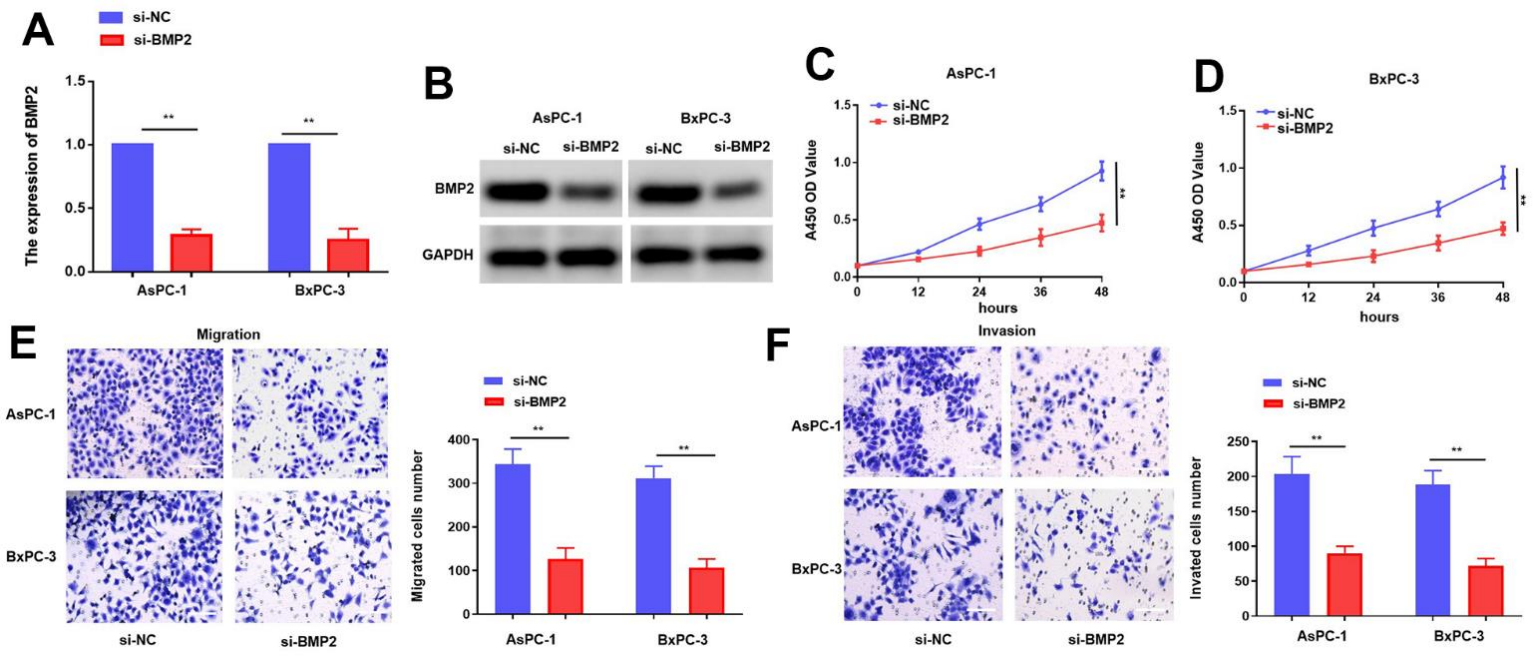
**BMP2 inhibits PC cells proliferation and metastasis while it is knocking down.** (**A**, **B**) The mRNA and protein of BMP2 affected by si-BMP2 siRNAs in PC cells. (**C**, **D**) CCK-8 assay with si-BMP2. (**E**, **F**) Transwell assay with si-BMP2 in PC cells. *p < 0.05 and **p < 0.01, scale bar, 100 μm.

Given the well-established oncogenic role of BMP2, our study aims to validate its functional significance in PC cells. The efficiency of BMP2 siRNAs in suppressing gene expression was evaluated in AsPC-1 and BxPC-3 cells through transfection, followed by analysis using qRT-PCR and western blotting [17]. The transfection of si-BMP2 resulted in a notable decrease in the levels of BMP2 in AsPC-1 and BxPC-3 cells (Figure 5A, 5B). Moreover, we further investigated the effects of si-BMP2 on the proliferative and metastatic potential of PC. The outcomes obtained from the CCK-8 assay revealed a notable reduction in proliferation rates for both AsPC-1 and BxPC-3 cell lines transfection with si-BMP2 (Figure 5C, 5D). Furthermore, the transwell assay revealed a reduction in migration activity of AsPC-1 and BxPC-3 cells following si-BMP2 transfection when compared to the control group. (Figure 5E, 5F). The combined results of our investigation suggest that the suppression of BMP2 activity leads to a decrease in both growth and metastasis.

### MiR-211-5p inhibits the progression of PC via BMP2

**Figure 6:**
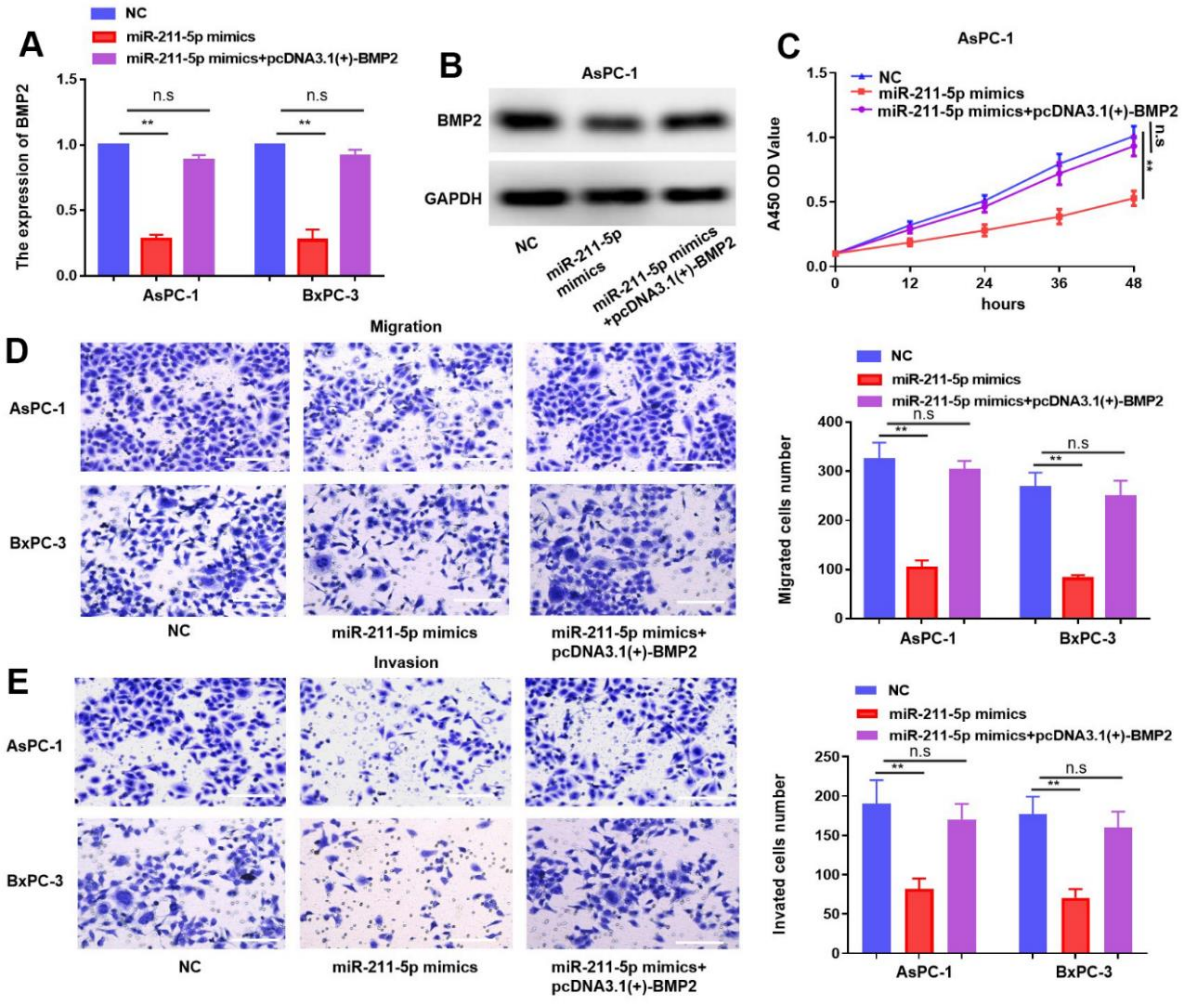
**MiR-211-5p effect rescued by the overexpression of BMP2 in PC.** (**A**, **B**) Expression of mRNA and protein of BMP2 in PC cells under transfection of miR-211-5p mimics and plasmid of pcDNA3.1(+)-BMP2. (**C**) Cell proliferation in PC cells under transfection of miR-211-5p mimics and pcDNA3.1(+)-BMP2 plasmid. (**D**, **E**) Transwell assay for PC cells migration under transfection of miR-211-5p mimics and pcDNA3.1(+)-BMP2. **p < 0.01, scale bar, 100 μm.

To elucidate the pivotal role of BMP2 in orchestrating regulation of miR-211-5p, we transfected miR-211-5p mimics or miR-NC mimics into PC cells together with pcDNA 3.1(+)–BMP2 through transfection. The results obtained from qRT-PCR and western blotting revealed a significant reduction in the abundance of BMP2 caused by miR-211-5p, which was partly reversed by BMP2 (Figure 6A, 6B). The CCK-8 assay revealed that the inhibitory impact of miR-211-5p on cellular proliferation in AsPC-1 and BxPC-3 cells was counteracted by BMP2 overexpression (Figure 6C). Additionally, the transwell assay also revealed that BMP2 upregulation attenuated the inhibitory effect of miR-211-5p on cellular metastasis in AsPC-1 and BxPC-3 cells (Figure 6D, 6E). Our findings suggest that the modulation of BMP2 plays a significant role in mediating the regulatory effects of miR-211-5p on the proliferative and metastatic of PC cells.

## DISCUSSION

Accumulating evidences has showed that miRNAs serve as a crucial role as regulators in the progression of oncogenesis and are recently extensively studied in the development of PC [26, 27]. Dysregulation of miRNAs plays a role in the progression of PC by initiating cell growth signaling and promoting the upregulation of genes associated with metastasis [28, 29]. Firstly, it is recommended that studies of miR-211-5p in other cancers could be sufficiently discussed. However, there has been a scarcity of relevant research investigating the functional of miR-211-5p in PC. Our investigation unveiled a significant reduction in the levels of miR-211-5p in PC, its presence was discovered to be associated with a favorable prognosis among individuals diagnosed with PC. Wang et al. it was discovered that bladder cancer exhibited a notable decrease in miR-211-5p, which had the ability to suppress bladder metastasis through its targeting of HDAC9 [30]. The results obtained from their investigation, in conjunction with our own clinical observations, revealed that miR-211-5p possessed the capability to impede the advancement of PC. Furthermore, in function assays, we noticed a notable suppression of the proliferative and metastatic abilities of PC cells when miR-211-5p was overexpressed. Our findings have revealed the significant role played by miR-211-5p in the progression of PC.

To further elucidate its underlying molecular mechanisms, we then study the potential functional mechanisms of miR-211-5p in PC cells by investigating the downstream targets. Based on the comprehensive analysis conducted using bioinformatics tool TargetScan and incorporating findings from previous studies [31]. We placed emphasis on bone morphogenetic protein 2 (BMP2), which has been found to regulate bone and cartilage formation [32, 33]. Although the relationship between BMP2 and various tumors, as well as the underlying mechanism of action, has been previously described, the impact of BMP2 on PC development remains elusive [34, 35]. The bioinformatics database analysis revealed BMP2 as the potential gene targeted by miR-211-5p. Further investigation using luciferase gene detection technology confirmed the specific binding of miR-211-5p to a target site located on the 3’untranslated region (3’UTR) of BMP2. Moreover, the mRNA and protein expression of BMP2 was detected in cells transfected with miR-211-5p mimics, confirming that miR-211-5p specifically targets BMP2 gene. The regulation of BMP2 was found to be influenced by miR-211-5p at the post-transcriptional level. In the present study, we discovered that BMP2 was a target of miR-211-5p.

In summary, our results indicate that the functionality of BMP2 in PC cells is directly affected by miR-211-5p through targeted regulation. Interestingly, we found that their expression levels in PC tissues were negatively correlated. Reduced expression of miR-211-5p was associated with a decrease in survival time among PC patients. Furthermore, rescue experiments demonstrated that the progression of PC is facilitated by miR-211-5p specifically targeting BMP2. These findings suggest that targeting miR-211-5p could be a promising approach for future therapeutic interventions in PC.

## CONCLUSIONS

Our research results indicate that the inhibitory effect of pancreatic cancer cell proliferative and metastatic is significantly influenced through miR-211-5p, which interacts with BMP2. This discovery suggests that miR-211-5p may be a prognostic biomarker for PC. However, further investigation was needed to found other downstream targets regulated by miR-211-5p and explored its regulatory mechanism in cancer.
